# Linking Biological Parameters to Fishery Management: Stock Assessment of Green Tiger Prawn, *Penaeus semisulcatus* De Haan, 1844 Along the Red Sea Coast of Saudi Arabia

**DOI:** 10.3390/biology15010008

**Published:** 2025-12-19

**Authors:** Eyüp Mümtaz Tıraşın, Sheeja Gireesh, Sirajudheen Thayyil Kadengal, Ronald Grech Santucci, Zahra Okba, Santhosh Kumar Charles, Goutham Bharathi Muthu Palani, Adel M. S. Adam, Mark Dimech

**Affiliations:** 1 KAUST Beacon Development Department, National Transformation Institute, King Abdullah University of Science and Technology, Innovation Cluster, 4700, Thuwal 23955-6900, Saudi Arabia; mumtaz.tirasin@deu.edu.tr (E.M.T.); sheeja.gireesh@kaust.edu.sa (S.G.); sirajudheen.kadengal@kaust.edu.sa (S.T.K.); ronald.grechsantucci@kaust.edu.sa (R.G.S.); santhosh.charles@kaust.edu.sa (S.K.C.); goutham.muthupalani@kaust.edu.sa (G.B.M.P.); adel.adam@kaust.edu.sa (A.M.S.A.); mark.dimech@kaust.edu.sa (M.D.); 2 Institute of Marine Sciences and Technology, Dokuz Eylül University, Balçova 35340, İzmir, Türkiye

**Keywords:** crustacean fisheries, shrimp trawling, length frequency, growth, mortality, maturity, stock assessment, spawning potential ratio, overfishing

## Abstract

*Penaeus semisulcatus* is the dominant commercial prawn species along the Saudi Arabian coast in the southeastern Red Sea, yet its population dynamics remain poorly understood. This study examined growth, maturity, and mortality using fishery-independent samples obtained during trawl surveys off Jizan and Al Qunfudhah between October 2022 and September 2023. A total of 85,909 individuals were examined, exhibiting carapace lengths (*CL*) between 1.29 and 56.14 mm and weights (*W*) ranging from 0.91 to 94.99 g. The sex ratio (1:1.06) was slightly male-biased. The *CL*–*W* relationships were *W* = 0.00427·*CL*^2.50^ for females and *W* = 0.01274·*CL*^2.16^ for males. The von Bertalanffy growth parameters were *CL*_∞_ = 60.16 mm, *K* = 1.03 year^−1^ for females and *CL*_∞_ = 48.10 mm, *K* = 1.02 year^−1^ for males. Females first matured at a *CL* of 22.09 mm. Exploitation rates (0.63 for females and 0.69 for males) and spawning potential ratio analysis indicated severe overfishing, with spawning stock biomass reduced to 19% of its unexploited level. These results highlight the necessity for immediate management intervention. Reducing fishing effort by half, extending seasonal closures, and improving the selectivity of trawl gear are advised to facilitate stock recovery and support sustainable exploitation in the region.

## Simple Summary

Penaeid shrimps, highly prized in global seafood markets, form the main target of marine shrimp fisheries. Their ecological and commercial importance underscores the necessity of conducting robust stock assessments to guide and ensure sustainable management. The present study investigates important population characteristics of the green tiger prawn *Penaeus semisulcatus* along the southeastern Red Sea coast. Growth analysis showed that the asymptotic carapace length is higher in females than in males, with both sexes exhibiting rapid growth rates. The estimated exploitation rates exceeded the optimum level for both sexes, raising concerns about the sustainability of the shrimp trawl fishery. Notable presence of juvenile prawns in the catch, coupled with excessive fishing mortality, strongly suggests that the stock is overexploited. To mitigate this situation, a transition from traditional diamond mesh codends to square meshes with improved selectivity is recommended to decrease juvenile catches and enhance sustainability. Additional measures, including limiting fishing days, curbing the active trawling fleet, setting catch limits, and prolonging seasonal closures, should be implemented to protect spawning and recruitment periods. Strengthening data collection for ongoing monitoring, incorporating climate-related factors into management, and integrating all actions into an ecosystem-based management framework created in partnership with key stakeholder groups are essential to ensure the long-term sustainability of shrimp stocks and fisheries.

## 1. Introduction

*Penaeus semisulcatus* De Haan, 1844, commonly known as the green tiger prawn, is a widely distributed penaeid shrimp species native to the Indo-West Pacific region. Its natural range spans from the Red Sea and East Africa, across the Arabian Gulf, the Indian subcontinent, and Southeast Asia, through the Malay Archipelago, and eastward to northern Australia and the northwestern Pacific. Notably, the species has successfully migrated through the Suez Canal into the eastern Mediterranean, where established populations now occur along the coasts of Egypt, Israel, Lebanon, Syria, and southern Türkiye [1,2,3]. In several regions, including the Gulf of Aden, the Arabian Gulf, and the coastal waters of India and Pakistan, *P*. *semisulcatus* supports important commercial offshore fisheries. It is also reported to be of commercial importance in Sri Lanka, Singapore, and the Philippines [4].

*P*. *semisulcatus* typically inhabits muddy and sandy bottoms at shallow to moderate depths and is commonly found in tropical and subtropical marine environments [1,5]. The species is gonochoristic and exhibits pronounced sexual dimorphism, with females reaching a maximum total length of 251 mm, while males can grow up to 210 mm [4]; the maximum recorded individual weight is 146.7 g [6]. It has a relatively short lifespan, typically completing its life cycle within 2 years [7,8,9]. The biology, ecology, and fishery dynamics of this species have been the focus of several studies, particularly in the Arabian Gulf and the Arabian Sea [6,10,11,12,13,14,15,16,17], including research conducted along the Saudi Arabian coast of the Arabian Gulf [18,19]. In contrast, the population dynamics of *P*. *semisulcatus* in the Red Sea have received comparatively little attention. The few existing studies include research from the southern Red Sea coast of Saudi Arabia [20,21,22,23,24] and the Yemeni coast [25].

Shrimp fishing grounds are located in the southern part of the Red Sea, where productivity surpasses that of the north [26], and the shrimp fishery in this area is primarily based on *P*. *semisulcatus* [20,22]. Catch records from the Saudi Arabian Ministry of Environment, Water, and Agriculture (MEWA) show that the total shrimp landings, encompassing all commercial species, from the Red Sea coast from 2017 to 2023 ranged between 500 and 835 tonnes, averaging 620 tonnes [27]. Previous studies in the region have reported that *P*. *semisulcatus* accounted for more than 80% of the total shrimp catch [21,24,28]. Despite its commercial relevance, comprehensive stock assessments remain limited, leaving a substantial knowledge gap in the management of regional fisheries. Understanding its growth, mortality, and reproductive potential under current exploitation levels is crucial for supporting sustainable harvest strategies. This study addresses this gap by investigating the population dynamics and stock status of *P*. *semisulcatus* along the southeastern coast of the Red Sea. It provides baseline biological parameters and exploitation indicators to inform science-based management of this key fishery resource.

## 2. Materials and Methods

### 2.1. Study Area and Biological Data Collection

**Figure 1:**
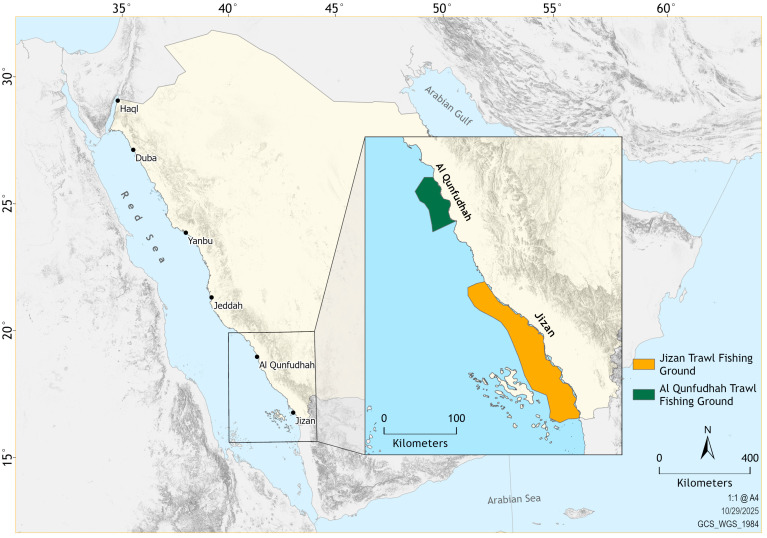
Locations of sampling sites in the trawl fishing grounds off Al Qunfudhah and Jizan along the southeastern Red Sea coast of Saudi Arabia.

Shrimp trawling along Saudi Arabia’s Red Sea coastline is legally restricted to two ports, Al Qunfudhah and Jizan (Figure 1), and only industrially licensed fishing vessels are permitted to operate from these ports. The vessels are typically wooden, often reinforced with fiberglass or metal plating [29]. National fishery regulations [30] require that all such vessels be equipped with modern navigational and safety systems, including GPS, sonar, communication devices, refrigeration units, and winches. Vessel specifications are capped at a maximum overall length of 20 m and engine power not exceeding 250 HP. Licensing is administered by the MEWA, which governs both the total number of active vessels and the conditions under which licenses are granted, including those issued to investor fishers who may own vessels but are not personally engaged in fishing. The fishery is subject to seasonal and spatial management measures, including a closed season from April to August, a two-nautical-mile no-trawl buffer zone from shore and islands to protect coastal habitats, and a minimum codend mesh size of 38 mm [30]. Among the two ports, Jizan, situated near the Farasan Islands, functions as the primary shrimp landing center, supporting a fleet of 151 registered industrial trawlers, compared to 24 in Al Qunfudhah [29,31]. Trawling operations in Jizan generally follow an oblique path relative to the coastline, targeting depths of 10–40 m, whereas trawling in Al Qunfudhah is typically oriented parallel to the shore at depths of 10–30 m. Trawling grounds in both regions consist predominantly of sandy bottoms, with scattered muddy patches [31,32]. Although the focus remains on trawling, some vessels switch to purse seining during the closed season of the shrimp fishery [29].

Between October 2022 and September 2023, monthly sampling was conducted in offshore waters near Jizan and Al Qunfudhah (Figure 1). Sampling was carried out using chartered industrial shrimp trawlers registered in the two ports, under the auspices of the MEWA. These vessels operated under standardized protocols designed to replicate commercial fishing practices with respect to gear type, haul duration, fishing locations, and operational procedures. Trawling was conducted exclusively at night, and the duration of each haul was about three hours at a towing speed of around three knots. Although weather conditions occasionally affected sampling frequency, a mean of 9 hauls was completed monthly at each site. During the survey period, 108 hauls were conducted off Jizan and 108 in Al Qunfudhah. All trawling operations employed the regionally used 40 mm diamond mesh codend [29,32]. A special exemption from the MEWA authorized sampling during the official fishery closure period from April to August.

After each trawl was brought onboard, all shrimps were separated from the rest of the catch and placed on ice in individual containers for transport to the laboratory. In the laboratory, specimens were sorted by species, and all *P*. *semisulcatus* individuals were selected for detailed examination. For each specimen, carapace length (*CL*) was measured to the nearest 0.01 mm using a digital caliper (Figure S1), and total weight (*W*) was recorded to the nearest 0.01 g. Sex was determined macroscopically based on the presence of thelycum in females or a petasma in males [9].

Gonadal maturity was assessed only in female shrimps, following a modified methodology adapted from Niamaimandi et al. [15] and Rajkumar et al. [33]. Ovarian development was classified into four distinct maturity stages based on ovary size and coloration: Stage I—Immature or undeveloped, Stage II—Developing, Stage III—Mature, and Stage IV—Spent (Table S1, Figure S2).

### 2.2. Data Analysis

#### 2.2.1. Carapace Length–Weight Relationship

Sex-specific differences in mean CL and W were tested using independent *t*-tests [34]. Before performing the main analyses, the data were examined to verify compliance with the assumptions of normality and homoscedasticity. For this purpose, a single-factor analysis of variance (ANOVA) was fitted separately for CL and W, with sex as the explanatory variable. The residuals from these ANOVAs were then assessed for normality using quantile–quantile plots and for homogeneity of variances using Levene’s test [34]. When variances were unequal, but normality was satisfied, Welch’s *t*-test [35] was employed. In cases where both assumptions were violated, a general Box–Cox transformation [35] was applied to stabilize variance and improve normality.

The relationship between *CL* and *W* was described using the nonlinear allometric model [36,37]:

(1) $$W=a\cdot {CL}^{b}$$

Here, *a* is the coefficient of proportionality, and *b* is the allometric exponent that reflects how *W* changes with *CL*. The parameters *a* and *b* were estimated by linear regression after natural log-transforming all data pairs, assuming a multiplicative error structure [36]. To test for sex-specific differences in growth, analysis of covariance (ANCOVA) was applied with sex as a factor [34]. Departures from isometric growth were assessed by testing whether the estimated *b* significantly deviated from the theoretical value of 3. This was evaluated by examining whether the 95% confidence intervals (CIs) constructed for *b* included 3 [36,38].

#### 2.2.2. Sex Ratio and Carapace Length at First Maturity

The overall sex ratio was first evaluated against the expected 1:1 proportion using an exact binomial test [39]. To test whether the sex ratio varied among months, a log-likelihood ratio test (*G*-test) was applied [35]. When the *G*-test indicated significant monthly variation, exact binomial tests were subsequently conducted for each month to determine whether the observed ratios deviated from parity. The *CL* at first sexual maturity (*CL*_50_), defined as the *CL* at which half of the individuals in the population are mature, was derived for females based on a logistic regression model [40], following the method of Aydın and Tıraşın [41]. Female maturity status was treated as a binary variable, with Stage I gonads classified as immature (=0) and Stage II or higher as mature (=1). These binomial data were then analyzed with the logistic model to obtain the *CL*_50_. To assess the precision of this estimate, 5000 bootstrap resamples were generated, and the bias-corrected and accelerated method [41,42] was employed to construct a nonparametric 95% CI.

#### 2.2.3. Growth

The growth of *P*. *semisulcatus* was modeled using the seasonally oscillating version of the von Bertalanffy [43] growth model [44]:

(2) $${CL}_{t}={CL}_{\infty }\cdot \left(1-e^{-K\cdot \left(t-t_{0}\right)-\left(\frac{C\cdot K}{2\pi }\right)\cdot (sin\left(2\pi \cdot \left(t-t_{s}\right)\right)-sin\left(2\pi \cdot \left(t_{0}-t_{s}\right)\right))}\right)$$

In this model, *CL_t_* represents the expected *CL* at age (*t*) years, *CL_∞_* is the asymptotic *CL*, *K* is the growth coefficient (or curvature parameter) that defines the shape of the growth curve, i.e., how quickly *P*. *semisulcatus* approaches its *CL*_∞_. The parameter *t*_0_ denotes the hypothetical age at which *CL* is zero. The parameter *C* specifies the amplitude of seasonal oscillations in growth, while *t_s_* indicates the position within the year at which the seasonal growth cycle begins, measured relative to *t*_0_ [44,45]. No seasonal oscillation occurs when *C* equals zero, and the model in Equation (2) simplifies to the traditional von Bertalanffy growth model as parameterized by Beverton and Holt [46].

The seasonally oscillating von Bertalanffy growth model parameters, with the exception of *t*_0_, were estimated separately for females and males using the TropFishR package (version 1.6.5; Mildenberger et al. [47]; Taylor and Mildenberger [48]. This package implements the electronic length-frequency analysis (ELEFAN) method [45,49]. Because true age (*t*) is required as the predictor variable in the von Bertalanffy growth model, and length frequency data only reflect the timing of sample collection, *t*_0_ cannot be reliably estimated from such data alone [45]. TropFishR, however, offers the parameter “*t_anchor_*”, which specifies the annual fraction (from 0 to 1) when a cohort’s growth curve intersects zero *CL* [47]. Parameter estimation was carried out using the ELEFAN_GA function, a genetic algorithm-based optimization procedure that simultaneously searches across all parameters. Input data consisted of monthly *CL* frequency distributions for each sex, grouped into 2 mm bins. Prior to curve fitting, the *CL* frequency data were restructured using the “lfqRestructure” function with a moving average of 5 bins, a commonly recommended setting for moderate bin widths [48]. A comparison of growth performance between females and males was carried out using the growth performance index (*φ′*) of Pauly and Munro [50], which integrates the *CL*_∞_ and *K* into a single comparative metric:

(3) $$\varphi^{\prime }={log}_{10}\left(K\right)+{2\cdot log}_{10}\left({CL}_{\infty }\right)$$

#### 2.2.4. Mortality

A linearized length-converted catch-curve analysis [51] was used to estimate the total mortality rate (*Z*) for males and females separately. Following the recommendations of Quinn and Deriso [36], Kenchington [52], Cope and Hamel [53], and Maunder et al. [54], the natural mortality rate (*M*) for *P*. *semisulcatus* was calculated as the average of three empirical methods, two proposed by Hamel and Cope [55] and one by Then et al. [56]. This approach, rather than reliance on a single estimator, was adopted to reduce potential bias and increase robustness of the mortality estimate. The three estimators were expressed as follows:

(4) M=5.4tmax

(5) M=1.55·K

(6) $$M=4.118\cdot K^{0.73}\cdot {CL}_{\infty }^{-0.33}$$

*t_max_* in Equation (4) denotes the species’ maximum observed age, or its longevity. For *P*. *semisulcatus*, *t_max_* was assumed to be 2 years, based on previous studies [7,8,9]. *K*, included in both Equations (5) and (6), and *CL_∞_*, present in Equation (6), are the von Bertalanffy growth parameters previously described. All three methods were recently endorsed in a comprehensive review by Maunder et al. [54], which critically evaluated and compared existing approaches for estimating *M* in stock assessment studies. Because *Z* is the sum of fishing mortality (*F*) and *M* [36,51], once estimates of *M* and *Z* were available, *F* was calculated as follows:

(7) F=Z−M

#### 2.2.5. Stock Status Evaluation

To evaluate the sustainability of current fishing practices along the southeastern Red Sea coast, a preliminary stock assessment of *P*. *semisulcatus* was performed using exploitation rate (*E*), yield-per-recruit (YpR), and spawning potential ratio (SPR) analyses. *E* was computed separately for males and females by expressing *F* as a proportion of Z, as presented below [36,51]:

(8) E=FZ

SPR is a primary indicator of a stock’s reproductive capacity under current fishing pressure, focusing on females as the source of egg production and recruitment. It is defined as the equilibrium spawning stock biomass (SSB) per recruit (SSBpR) at a given *F*, divided by the SSBpR in the absence of fishing [57]. SPR analysis extends traditional yield- and biomass-per-recruit (YpR and BpR) models formulated by Beverton and Holt [46] and Thompson and Bell [51]. Similarly to BpR, SSBpR quantifies the biomass contributed by each recruit, but only mature, spawning females are considered. SPR equals 1 (or 100%) when *F* is 0 and monotonically decreases toward 0 as *F* rises. Thus, SPR quantifies the fraction of the unfished reproductive potential retained by the stock under the specified level of *F*.

To obtain SPR estimates for female *P*. *semisulcatus*, a length-based Thompson and Bell YpR model [47,51] was utilized. Females were the focus of the analysis, as they are directly responsible for egg production and therefore strongly influence the population’s overall reproductive potential. The YpR calculations incorporated the parameters *M*, *CL_∞_*, and *K* for each sex, together with the number of individuals and their mean weight in each 2 mm *CL* class, consistent with the data used for estimating the von Bertalanffy growth parameters. The *CL* at first capture (*CL_c_* = 14 mm), derived from Santucci et al. [32], a recent study that used commercial shrimp trawl gear off Jizan and Al Qunfudhah, was also incorporated as an input. The same *M* value was applied to calculate survivors across all *CL* classes. For *CL* classes at *CL_c_* and above, calculations incorporated both *F* and *M* values. The *F* array used in the model ranged from 0 to 5 year^−1^ in 0.01 increments. Maturity proportions for females within each *CL* class were obtained from the fitted logistic regression model. YpR, BpR, and SSBpR were then calculated across varying values of the *F* array and reported in units of weight (g).

Biological reference points (BRPs) obtained from the YpR model are specified as *F*-based mortality thresholds used to indicate stock status [36]. Two key BRPs, *F_max_* and *F*_0.1_, were estimated from the YpR curve. *F_max_* represents the *F* that yields the maximum YpR and is regarded as a limit reference point (LRP), while *F*_0.1_, defined as the *F* at which the YpR curve’s slope declines to 10% of its initial value, serves as a precautionary target reference point (TRP). The LRP marks a threshold beyond which overfishing may occur, whereas the TRP represents a sustainable *F* level that maintains stock productivity and long-term yield [36,58,59].

YpR analysis primarily assesses growth overfishing but does not account for the effects of fishing on reproductive capacity [36,60,61]. In contrast, SPR analysis evaluates recruitment overfishing by assessing the extent to which SSB remains sufficient to support future recruitment [36,62]. An SPR of 0.4 is widely recommended as the TRP corresponding to *F*_40%_, while 0.3 (*F*_30%_) is often adopted as the LRP to avoid recruitment failure, given the elevated risk of overfishing at lower values [28,57,60,63].

In addition to SPR-based benchmarks, an *E* value of 0.5 was adopted as the LRP, denoting the upper boundary of sustainable fishing pressure beyond which overexploitation is likely to occur. This criterion aligns with the principle that *F* should not surpass *M*, expressed as *F*/*M* ≤ 1 [64,65], and serves as an indicator of the risk of overexploitation for *P*. *semisulcatus* in the region.

All computations, statistical analyses, and graphical visualizations were performed using R software version 4.5.1 [66], with a 5% significance level applied to all statistical tests.

## 3. Results

### 3.1. Distribution of the Samples

During the study period, 103,758 individual shrimp representing six commercially important species were sampled in the study area. The species composition was heavily dominated by *P*. *semisulcatus*, which comprised the vast majority of specimens (*n* = 85,909, 83%). Second in abundance was *Metapenaeus monoceros*, accounting for a moderate 10% (*n* = 10,859) of the total catch [28]. The remaining four made only minor contributions: *Penaeus pulchricaudatus* (*n* = 3274) and *Penaeus indicus* (*n* = 2860) each comprised approximately 3%, while *Penaeus hathor* and *Penaeus monodon* were comparatively rare, representing only 1% (*n* = 839) and 0.02% (*n* = 17) of the total, respectively. This marked disparity highlights *P*. *semisulcatus* as the predominant species in the fishery and the most abundant commercially exploitable stock in the surveyed region. With respect to sampling locations, Jizan yielded the majority of *P*. *semisulcatus*, 51,365 individuals (60%), whereas Al Qunfudhah contributed 34,544 individuals (40%).

### 3.2. Sex Ratio and Maturity

**Figure 2:**
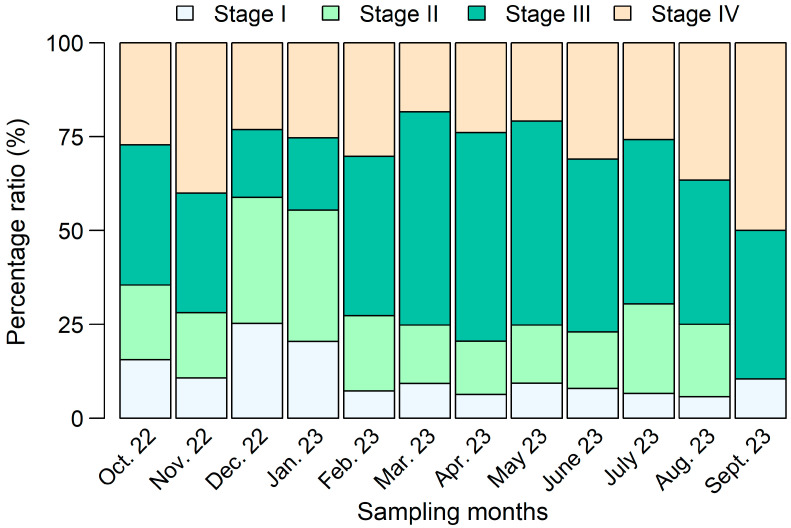
Monthly variation in the percentage composition of female *Penaeus semisulcatus* maturity stages in the southeastern Red Sea.

Of the 85,909 *P*. *semisulcatus* specimens examined, 41,657 were females, and 44,225 were males, while 27 individuals could not be sexed. The overall sex ratio showed a slight but statistically significant predominance of males (F:M ≈ 1:1.06; binomial test, *p* = 0.016). However, the ratio varied significantly across months (log-likelihood ratio test, *G* =762.1, df = 11, *p* < 0.001). In December 2022, April 2023, and September 2023, the F:M ratio did not differ from the expected 1:1 proportion. Females outnumbered males only in February 2023; in all other months, the sex ratio deviated significantly from unity in favor of males (Figure S3). Figure 2 illustrates the monthly percentage distribution of maturity stages for 40,393 female *P*. *semisulcatus*. Females representing all maturity stages were present in nearly every sampling month, except for Stage II, which was absent in September 2023. Stage III females (*n* = 15,992) were most prevalent from February to July 2023, reaching their highest monthly representation (57%) in March 2023. The minimum recorded *CL* for Stage III females was 18.61 mm, and their average *CL* (±SD) was 33.45 ± 5.31 mm.

**Figure 3:**
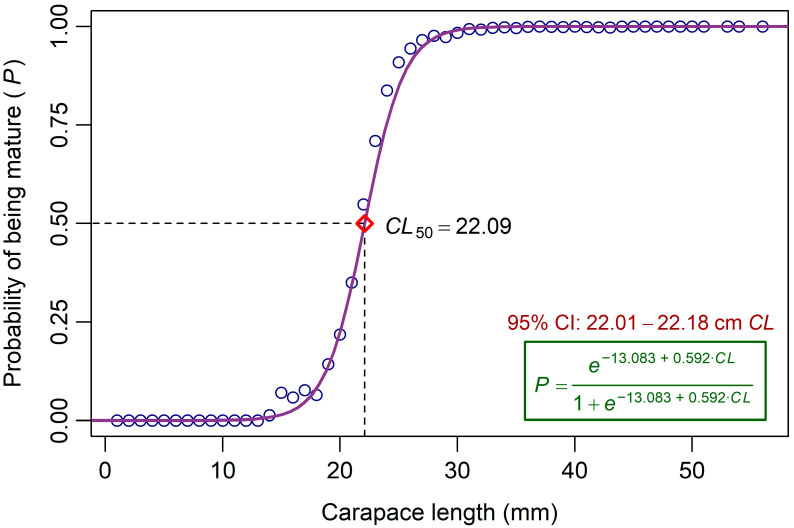
Estimated carapace length at first sexual maturity (*CL*_50_) of female *P*. *semisulcatus* from the southeastern Red Sea. The curve represents the logistic model fitted to the binary maturity data. Circles indicate the observed proportions of mature specimens relative to the total number of sampled females within each 1 mm *CL* class. The red diamond symbol marks the estimated *CL*_50_ on the logistic curve. CI denotes the confidence intervals.

**Figure 4:**
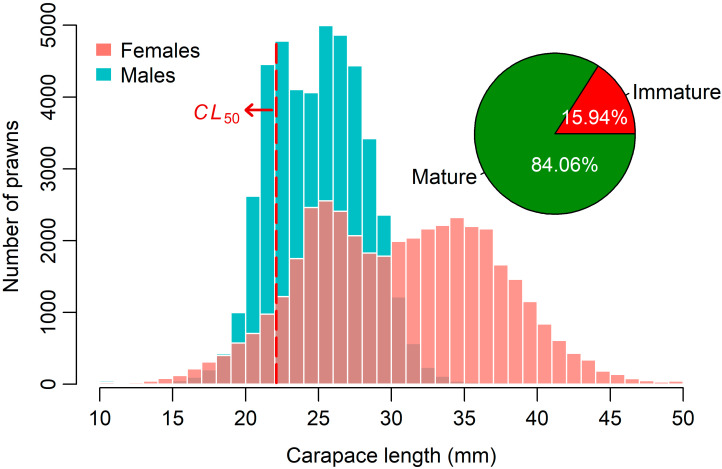
*CL* frequency distribution of female and male *P*. *semisulcatus* sampled from the southeastern Red Sea. Female bars are plotted in the foreground with partial transparency, so overlapping areas appear darker where male and female distributions intersect. *CL*_50_ denotes the *CL* at first sexual maturity for females, estimated at 22.09 mm. The proportions of immature (*CL* < *CL*_50_) and mature (*CL* ≥ *CL*_50_) prawns in the combined overall sample are also illustrated.

The *CL*_50_ estimate for females was 22.09 mm, with a 95% CI of 22.01–22.18 mm (Figure 3). Although *CL*_50_ was estimated only for females, the same threshold was applied to the total catch (females and males combined) solely as a reference indicator to quantify the proportion of *P*. *semisulcatus* harvested below the size at which females mature. Based on the overall *CL* distribution of all specimens collected during the study period, immature prawns (*CL* < *CL*_50_) represented about 16% of the total samples, while mature individuals (*CL* ≥ *CL*_50_) accounted for just over 84% (Figure 4).

### 3.3. Carapace Length Frequency Distribution

Across the entire sample of *P*. *semisulcatus*, *CL* varied between 1.29 and 56.14 mm, whereas *W* ranged from 0.91 to 94.99 g. Table 1 summarizes the descriptive statistics for *CL* and *W*, including range, mean, standard deviation (SD), median, and interquartile range (IQR), reported separately by sex and for the combined sample, including the 27 unsexed specimens. Compared with males, females showed a consistently broader range for both *CL* and *W*, indicating they attain larger maximum sizes within the sampled population (Table 1). The variability (dispersion) in body size was also significantly greater in females, as reflected by the larger SD values for both *CL* and *W* (Levene’s test for *CL*, *F* = 12,770, df = 1, 85,880, *p* < 0.001; for *W*, *F* = 10,334, df = 1, 83,536, *p* < 0.001). Moreover, females demonstrated significantly greater mean *CL* and *W* compared with males (Welch’s approximate *t*-test for *CL*, *t* = 160.4, df = 61,204, *p* < 0.001; for *W*, *t* = 169.7, df = 61,204, *p* < 0.001). These statistically significant differences confirm pronounced sexual dimorphism in body size, with females being substantially larger and more variable in size than males within the studied population.

**Figure 5:**
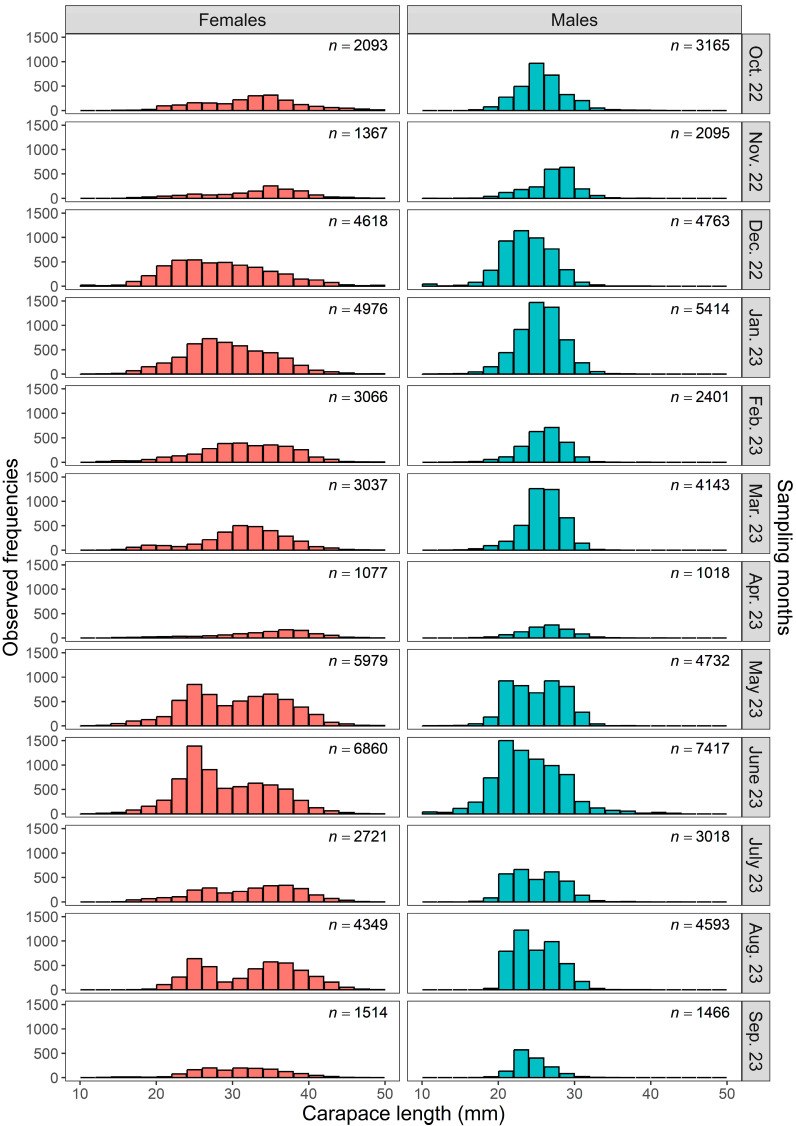
Monthly *CL* frequency distributions of female and male *P*. *semisulcatus* from the southeastern Red Sea, grouped into 2 mm class intervals.

The *CL* frequency distribution of the total sample of *P*. *semisulcatus* from the southeastern Red Sea, arranged by 1 mm class intervals and categorized by sex, is shown in Figure 4. Specimens outside the main size range (10–50 mm *CL*) were scarce; therefore, 7 females and 15 males with *CL* < 10.00 mm were included in the 10–11 mm class, while 13 females with *CL* > 50.00 mm were grouped into the 49–50 mm class to ensure continuous class intervals and adequate representation across the size range. All small individuals (*CL* 2–10 mm) were encountered exclusively in December 2022, which likely indicates the period of major recruitment. Additionally, the monthly *CL* frequency distributions of *P*. *semisulcatus*, separated by sex, are presented in Figure 5. Notable differences in *CL* distribution patterns between sexes were evident throughout the sampling period. Both the mean and median *CL* values of females remained consistently higher than those of males each month, and females also exhibited a broader monthly *CL* range, reflecting their tendency to attain larger sizes.

### 3.4. Carapace Length–Weight Relationship

**Figure 6:**
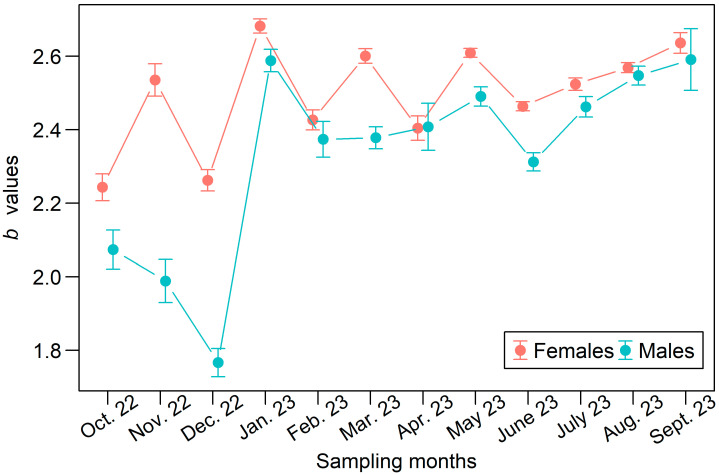
Estimated *b* values for the *CL*–*W* relationship of female and male *P*. *semisulcatus* in each sampling month. Vertical bars represent the associated 95% CIs.

The ANCOVA applied to monthly *CL* and *W* data revealed significant differences in the a and *b* parameters across certain months, as well as between females and males (ANCOVA: *F* = 1981, df = 47, 83,490, *p* < 0.001). The monthly variations in the estimated *b* values for each sex are presented in Figure 6. Females exhibited significantly higher *b* values than males in October, November, and December 2022, as well as in January, March, May, June, and July 2023 (Figure 6). In the remaining four months, the differences in *b* estimates between the sexes were not statistically significant. Despite these temporal variations, both sexes consistently displayed a negative allometric growth pattern throughout the study period. When all data were combined by sex, the estimated parameters (95% CIs) of the *CL*–*W* relationship were *a* = 0.00427 (0.00417–0.00437) and *b* = 2.50 (2.494–2.507) for females, and *a* = 0.01274 (0.01227–0.01323) and *b* = 2.16 (2.150–2.174) for males.

### 3.5. Growth and Mortality

Parameter estimates from the seasonally oscillating von Bertalanffy growth model were *CL*_∞_ = 60.16 mm, *K* = 1.03 year^−1^, *t_anchor_* = 0.53 years, *C* = 0.66, and *t_s_* = 0.68 years for females (Figure S4), and *CL*_∞_ = 48.10 mm, *K* = 1.02 year^−1^, *t_anchor_* = 0.33 years, *C* = 0.92, and *t_s_* = 0.48 years for males (Figure S5). The calculated growth performance indices were 3.57 for females and 3.37 for males. These results indicate that both sexes exhibit comparable *K* values, although females attain a larger asymptotic size, resulting in slightly higher overall growth performance compared to males.

The *Z* values (95% CIs), estimated from the linearized length converted catch curve analysis, were 5.96 (5.04–6.87) year^−1^ for females and 7.26 (6.01–8.53) year^−1^ for males, with a coefficient of determination (*r*^2^) of 0.94 for both regressions (Figure S6). The *M* estimates obtained from the three empirical methods showed considerable variation across methods but were relatively consistent between sexes. The method based on *t_max_* produced an estimate of 2.70 year^−1^ for both sexes, while the approach using the von Bertalanffy growth model parameter *K* yielded 1.59 year^−1^ for both sexes. The third method, which incorporated both *K* and *CL_∞_*, produced *M* values of 2.32 year^−1^ for females and 2.51 year^−1^ for males. The average *M* (±SD) calculated across these methods was 2.21 ± 0.56 year^−1^ for females and 2.27 ± 0.59 year^−1^ for males. Using the sex-specific *Z* and mean *M* values, the resulting estimates of *F* were 3.75 year^−1^ for females and 4.99 year^−1^ for males.

### 3.6. Stock Status Evaluation

**Figure 7:**
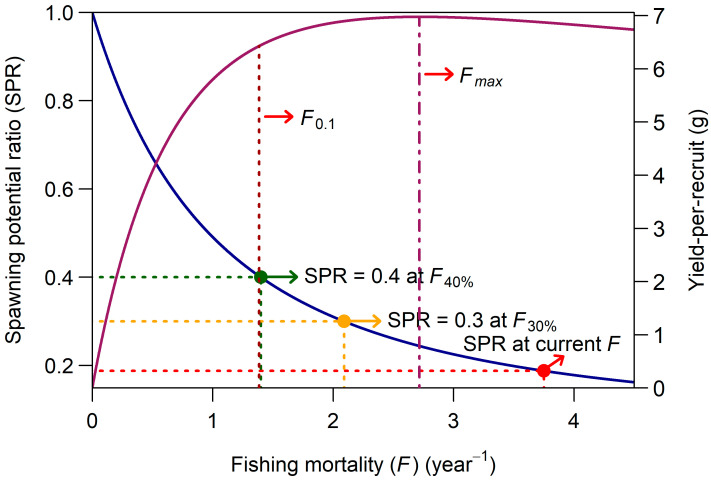
Relationship between spawning stock biomass per recruit (SSBpR; dark blue line, corresponding to the left Y-axis) and total yield-per-recruit (YpR; dark red line, corresponding to the right Y-axis) as a function of fishing mortality (*F*). The figure also shows the SPR values corresponding to the current *F* and key biological reference points, including *F_max_*, *F*_0.1_, *F*_40%_, and *F*_30%_.

The *E* levels estimated from sex-specific mortality parameters were 0.63 for females and 0.69 for males. Both estimates exceed the LRP of 0.5, which indicates that the *P*. *semisulcatus* is currently experiencing overexploitation in the southeastern Red Sea. Results of the Thompson and Bell YpR and SPR analyses for female *P*. *semisulcatus* under equilibrium conditions are presented in Figure 7. The left Y-axis shows the SPR, whereas the right Y-axis displays the total YpR for a single female prawn recruiting to the stock. The figure depicts the reduction in SSBpR relative to the unfished level and the corresponding changes in YpR across increasing values of *F*. *F_max_* was estimated to be 2.72 year^−1^, which yielded a maximum YpR of 6.98 g. Subsequent increases in *F* resulted in a steady decline in YpR. The precautionary TRP, *F*_0.1_, was 1.38 year^−1^. The current *F* for females (3.75 year^−1^, YpR = 6.86 g) markedly exceeded both *F_max_* and *F*_0.1_. The SSB at this level of *F* was reduced to nearly 19% of its unfished state. The SPR-based TRP *F*_40%_ was estimated as 1.40 year^−1^, closely matching the *F*_0.1_ estimate (Figure 7).

## 4. Discussion

Global marine fisheries are experiencing a marked decline, with particularly severe impacts on developing countries, primarily due to overfishing, pollution, increasing market demand, and inadequate management. Depletion of these resources threatens marine biodiversity, ecosystem stability, and the livelihoods of fishing communities. Effective strategies for recovery of stocks are, therefore, essential for achieving long-term ecological and economic sustainability. Science-based stock assessments and resource management remain critical instruments for safeguarding and sustainably managing living marine resources [67,68,69,70]. The present study offers updated and detailed information on the growth, maturity, mortality, and stock status of *P*. *semisulcatus* in the southeastern Red Sea, enabling cross-regional comparisons with previous investigations.

The trawl surveys in the southeastern Red Sea revealed that *P*. *semisulcatus* was the predominant species, accounting for 83% of the total shrimp count and 86% by weight. Abduallah et al. [21] reported similar observations, finding that this species dominated commercial trawl catches in the Jizan region, constituting 82–90% of the total shrimp catch during their study period. Consistent with these findings, Alsolami and Jastania [23] also reported that *P*. *semisulcatus* comprised the majority of the annual shrimp catch taken by bottom trawlers in the Jizan area, with relative abundance ranging from 84% to 93% between 2010 and 2016. A comparable dominance pattern was also noted by Sabry et al. [22], who reported this species as the principal component of shrimp catch in Jizan, although no quantitative estimates of abundance were provided. In contrast, Ghamrawy [20] found *P*. *semisulcatus* to be the most abundant species in Jizan, but accounting for a lower proportion, just over 53% of the total sampled shrimp. Variation in relative abundance reported by Ghamrawy [20] compared with more recent studies may be attributable to temporal changes influenced by environmental conditions, methodological differences among investigations, elevated fishing pressure, or broader alterations in ecosystem functioning.

The upper ranges of *CL* frequency distribution observed in this study were 56 and nearly 47 mm for females and males, respectively (Table 1). These values closely match those reported by Abdul-Wahab [25] from the Yemeni Red Sea, who recorded maximum *CL*s of 56 mm for females and 43 mm for males (Table 2). In contrast, Alsolami and Jastania [23] documented considerably larger individuals from the Jizan region on the Saudi Red Sea coast, with a maximum *CL* of 85 mm. Outside the Red Sea, most studies have reported larger maximum *CL*s for *P*. *semisulcatus*, such as 66 mm *CL* recorded by Suman et al. [71] from Indonesian waters (Table 2). Nevertheless, all these records remain below the exceptional size reported by Alsolami and Jastania [23]. The observed regional differences may be indicative of variations in environmental conditions, productivity, fishing intensity, or sampling methodologies across the species’ distribution range.

The *b* estimates obtained in the present study, 2.50 for females and 2.16 for males, indicate a negative allometric (hypoallometric) growth pattern, suggesting that in both sexes of *P*. *semisulcatus* increases in *CL* outpace gains in *W*. Estimates of the *CL*–*W* relationship parameters *a* and *b* reported in previous studies from the Red Sea and other regions are summarized in Table 2. Some of these studies provided sex-specific estimates, whereas others analyzed data for both sexes combined. The present findings are consistent with several studies that also reported negative allometric growth for this species in the Red Sea and elsewhere. For instance, Alsolami and Jastania [23] estimated a *b* value of 2.67 from the Jizan region, El-Ganainy and Yassien [72] reported 2.56 for the Egyptian Red Sea coast, and Suman et al. [71] obtained 2.50 for combined sexes from Indonesian waters. Similarly, Ragavan et al. [73] found a *b* value of 2.70 for the population in Sri Lanka. In contrast, Alrashada et al. [19], Mehanna [74], and Mohamed and El-Aiatt [75] reported isometric growth patterns for *P*. *semisulcatus* populations in the Egyptian Red Sea, the Egyptian Mediterranean, and the Arabian Gulf, respectively (Table 2). Moreover, Kumlu et al. [76] and Manaşırlı et al. [77] from the Turkish Mediterranean coast, and Mohamed et al. [6] and Hassan et al. [17] from the Iraqi coast of the Arabian Gulf, observed positive allometric (hyperallometric) growth in females, while males exhibited isometric growth (Table 2). Overall, the variability in *b* values across regions most likely reflects differences in environmental conditions, seasonality, food availability, reproductive stages, sampling methods, and sample size among the studies.

The sex composition of the overall samples leaned slightly towards males (F:M = 1:1.06), and although this deviation from parity was statistically significant, it still remained close to unity. This near 1:1 ratio, with a modest male bias, is consistent with findings from several other regions, including those reported by Alizadeh et al. [8], Alrashada et al. [19], Mustafa et al. [78], and Sarada [79]. In contrast, most studies (Table 2), including two conducted in the Red Sea, have documented sex ratios that are slightly skewed towards females in *P*. *semisulcatus* populations.

**Table 2:** Summary of maximum *CL* and *W*, sex ratio, *CL*–*W* relationship parameters, and *CL*_50_ of *P*. *semisulcatus* reported from different regions worldwide. F and M denote females and males, respectively. Values representing both sexes combined are placed between the F and M columns. Measurements based on total length (*TL*) rather than *CL* are marked with an asterisk (*). Studies using cm instead of mm in calculating *CL*–*W* relationship parameters are marked with two asterisks (**).

Location	Max *CL* (mm)		Max *W* (g)		Sex Ratio	*a*		*b*		*CL*_50_ (mm)		References
	F	M	F	M	(F:M)	F	M	F	M	F	M	
Saudi Arabia (Red Sea)	56	47	63	95	1:1.06	0.0043	0.0127	2.50	2.16	22		Present study
	85				1:0.91	0.0008		2.67				Alsolami and Jastania [24]
Saudi Arabia (Arabian Gulf)	62									23		Rabaoui et al. [18]
					1:1.18	0.0002		3.05				Alrashada et al. [19]
Yemen (Red Sea)	56	43				0.0024		2.70				Abdul-Wahab [25]
Yemen (Arabian Sea)						0.0079		2.37				Abdul-Wahab [80]
Kuwait (Arabian Sea)	56	38										Mohammed et al. [12]
Egypt (Red Sea)	244 *	202 *	136	69	1:0.91	0.0064	0.0067	3.09	3.07			Mehanna [74]
						0.4170		2.56				El-Ganainy and Yassien [72] **
[Oman (Arabian Sea)	61				1:0.85							Mehanna et al. [16]
Iraq (Arabian Gulf)	251 *		147			0.0044		3.21		145 *		Hassan et al. [17]
	251 *	201 *	147	74	1:0.94	0.0056	0.0068	3.14	3.03	142 *		Mohamed et al. [6]
Iran (Arabian Gulf)					1:0.84					24		Niamaimandi et al. [15]
	54	41			1:0.86	0.0077	0.0072	2.39	2.42	28		Alizadeh et al. [8]
	63	52			1:1.06	0.0071	0.0090	2.42	2.36	28		
Egypt (Mediterranean Sea)					1:0.68	0.1981	0.3321	2.98	2.54			Yassien [7] **
	62	50	63	24		0.0002	0.0003	2.99	2.87			Mohamed and El-Aiatt [75]
	165 *	161 *	32	31	1:0.93	0.0857	0.0390	2.15	2.50			Al-Beak et al. [81]
Türkiye (Mediterranean Sea)	220 *	170 *				0.0045	0.0072	3.25	3.04	36		Kumlu et al. [76]
	232 *	183 *	124	50	1:0.95	0.0064	0.0092	3.13	2.96			Manaşırlı et al. [77]
	250 *	190 *	136	56	1:0.73	0.0070	0.0120	3.05	2.87			Bayhan [82]
India (Arabian Sea)	220 *	190 *			1:1.07	0.0063	0.0059	2.99	3.02			Sarada [79]
	237 *	208 *			1:0.79					126 *	107 *	CMFRI [83]
India (Bay of Bengal)	260 *	215 *								135 *		CMFRI [83]
	251 *	210 *										Rajkumar et al. [4]
					1:0.74	0.0042	0.0064	3.27	3.07	114 *	100 *	Rajkumar et al. [84]
Bangladesh (Bay of Bengal)					1:1.20	0.0110	0.0116	2.92	2.89			Mustafa et al. [78]
Sri Lanka	48		57					2.70		27		Ragavan et al. [73]
Indonesia	66				1:0.93			2.50		39		Suman et al. [71]

The estimated *CL*_50_ for female prawns in this study was 22.09 mm. This result is consistent with the findings of Ghamrawy [20], who reported that *P*. *semisulcatus* in Jizan matured at similar sizes, with the smallest *CL* required to reach ovarian maturation Stages III and IV being 23 mm and 27 mm, respectively. Similarly, Rabaoui et al. [18] estimated a *CL*_50_ of 23 mm for female *P*. *semisulcatus* along the Saudi Arabian coast of the Arabian Gulf. Comparable values have also been reported in nearby regions of the Arabian Gulf, where *CL*_50_ estimates range from 24 to 28 mm, as documented by Niamaimandi et al. [15] and Alizadeh et al. [8], respectively (Table 2). Studies from other parts of the species’ distribution have shown either similar or higher *CL*_50_ values. Ragavan et al. [73] reported a *CL*_50_ of 27 mm in Sri Lanka, whereas larger sizes were observed in the Mediterranean (36 mm; Kumlu et al. [76]), Australia and Indonesia (39 mm; Crocos [85]; Suman et al. [71]), and the Bay of Bengal (40 mm; Rajkumar et al. [33]). In studies in which *TL* was used instead of *CL*, the maturity size ranged from 100 to 145 mm (Table 2). These variations in reported first maturity sizes likely reflect regional differences in environmental conditions, growth rates, reproductive cycles, and sampling methodologies across the species’ range.

The reproductive biology of *P*. *semisulcatus* has been extensively studied, revealing a pattern of year-round spawning activity, typically characterized by elevated reproductive output during specific seasons [8,17,71,77,80,83,86]. The temporal analysis of ovarian developmental stages identified in this study (Figure 2) similarly indicated that spawning occurs throughout the year, with a pronounced peak from March 2023 to July 2023 in the southeastern Red Sea. Comparable patterns have been reported in the Arabian Gulf, including Kuwait, where spawning peaks occur from May to August [12], and in Iraq, where a major spawning peak in May coincides with the onset of the warmer months [17]. Regional differences, however, are evident. Abdul-Wahab [80] recorded a contrasting peak between December and March along the Yemeni coast of the Arabian Sea, while Maheswarudu et al. [86] reported dual peaks (February–March and July–September) on the southeast coast of India. Such variability likely reflects environmental influences, particularly temperature, monsoon cycles, and food availability, underscoring the ecological adaptability of *P*. *semisulcatus* to diverse habitats. In terms of recruitment, Mohammed et al. [12] observed two recruitment pulses in the Arabian Gulf: a primary peak in June and July, and a secondary peak in August and September. The proportion of new recruits was highest between June and August, followed by a sharp decline thereafter. This recruitment pattern seems to closely correspond to the spawning cycle of the species. In Indonesia, Suman et al. [71] reported a peak in recruitment during the transition from the rainy to the dry season, when clear waters and cooler temperatures favor successful reproduction. In contrast, Hassan et al. [17] documented continuous recruitment throughout the year in Iraq, with a single prominent peak in May. In the present study, recruitment appeared less pronounced throughout the year; however, the occurrence of small individuals (*CL* 2–9 mm) exclusively in December 2022 suggests that a major recruitment event may occur during this period in the southeastern Red Sea. This observation provides additional evidence of the spatial and temporal variability in the reproductive and recruitment dynamics of *P*. *semisulcatus* across its range.

In the present study, the *CL*_∞_ of female *P*. *semisulcatus* was estimated to be 60.16 mm, whereas that of males was 48.10 mm. Comparable estimates have been documented in previous studies from the Red Sea region, including 58.5 and 58.8 mm for females and 44.9 and 44.6 mm for males from Jizan [22] and the Yemeni coast [25]. In contrast, the *CL*_∞_ estimate recorded by Alsolami and Jastania [23] in Jizan was substantially higher, reaching 94.4 mm for both sexes. In the Arabian Gulf, reported *CL*_∞_ values varied widely: lower estimates, such as 50.4 mm for females and 38.0 mm for males, were found by Niamaimandi et al. [14], whereas larger values, 69.0 mm for females and 55.0 mm for males, were recorded by Alizadeh et al. [8]. Studies that documented *TL*_∞_ estimates provided ranges of 221.5–293.2 mm for females and 197.2–263.0 mm for males. Table 3 summarizes the growth parameters of *P*. *semisulcatus* reported from different regions across its distribution. Despite differences in the measurement types used (*CL* or *TL*), all sex-specific estimates consistently demonstrated clear sexual dimorphism, with females attaining a larger asymptotic size than males, a pattern documented across multiple regions and studies (Table 3). The *K* estimates in this study were 1.03 year^−1^ for females and 1.02 year^−1^ for males, indicating nearly identical growth rates between the sexes. However, because females attain a higher *CL*_∞_, their overall growth performance was slightly greater than that of males. As shown in Table 3, *K* estimates for *P*. *semisulcatus* reported from different regions vary considerably, ranging from as low as 0.5 year^−1^ [17] to as high as 2.2 year^−1^ [14], both from the Arabian Gulf.

In this study, the estimated *Z* values were 5.96 and 7.26 year^−1^ for females and males, respectively. Conversely, Sabry et al. [22] and Alsolami and Jastania [23] presented lower *Z* estimates than those obtained in the present study from the same fishing grounds along the southeastern coast of the Red Sea in Saudi Arabia. Sabry et al. [22] found nearly similar rates for females (3.94 year^−1^) and males (3.60 year^−1^), while Alsolami and Jastania [23] recorded 3.12 year^−1^ for both sexes combined. The reported *Z* estimates for *P*. *semisulcatus* vary widely across regions; however, in most previous studies, males exhibited higher *Z* values than females, consistent with the present findings (Table 3). For instance, in the Arabian Gulf of Saudi Arabia, *Z* was 3.37 year^−1^ for females and 4.65 year^−1^ for males [19]. Comparable patterns were observed in Yemen, where Abdul-Wahab [25,80] derived higher *Z* values for males along both the Arabian Sea and Red Sea coasts. Specifically, *Z* was estimated at 5.63 year^−1^ for females and 6.55 year^−1^ for males from the Yemeni Red Sea, and at 5.6 and 7.3 year^−1^ for females and males, respectively, from the Arabian Sea. Similar trends have also been documented by Mustafa et al. [78], who reported slightly higher male mortality (5.20 year^−1^) than female mortality (4.70 year^−1^) in Bangladesh. Along the eastern and western Indian coast, *Z* values reported by CMFRI [83] were 8.91–9.00 year^−1^ for males and 7.50–8.45 year^−1^ for females, whereas Maheswarudu et al. [86] recorded the highest *Z* values from the eastern coast of India, as 13.93 year^−1^ for males and 8.14 year^−1^ for females. Similarly, Ye et al. [87], Alizadeh et al. [8], Yassien [7], and Bayhan [82] found markedly higher male mortality in Kuwait, Iran, Egypt, and Türkiye (Table 3). However, a few studies have reported the opposite trend. Villarta et al. [89] found lower *Z* values for males (3.61 year^−1^) than females (5.65 year^−1^) in the Philippines, while Mohamed and El-Aiatt [75] observed a similar pattern in the Egyptian Mediterranean (5.34 and 3.24 year^−1^ for females and males, respectively). Niamaimandi et al. [14], Mehanna et al. [16], Al-Beak et al. [81], and Rajkumar et al. [84] also documented higher female mortalities in Iranian, Omani, Egyptian, and Indian waters, respectively (Table 3).

The final *M* values, derived as the averages of estimates from three empirical methods, were 2.21 year^−1^ for females and 2.27 year^−1^ for males, with corresponding *F* estimates of 3.75 year^−1^ and 4.99 year^−1^, respectively. Although the sex-specific *M* values were nearly identical, the higher *Z* observed in males resulted in a higher *F* estimate, suggesting that males were subjected to more intense fishing pressure. The compiled *M* estimates for *P*. *semisulcatus* from different regions across its range (Table 3) show considerable variation, ranging from as low as 0.42 year^−1^ in the southeastern Mediterranean [81] to as high as 3.66 year^−1^ in the Bay of Bengal [73]. Most previous studies have provided similar *M* estimates for both sexes or slightly higher values for males (Table 3). In contrast, relatively few studies have documented the opposite pattern, with females exhibiting higher *M* values than males. Among these, Villarta et al. [89] from the Philippines stands out, reporting the largest sex-specific disparity in *M*, with estimates of 3.65 year^−1^ for females and 1.70 year^−1^ for males. Such variability in *M* estimates among studies likely reflects differences in environmental conditions, fishing pressure, and natural variability in growth and longevity among *P*. *semisulcatus* populations, especially within each sex and growth stage. In addition, the methodologies employed to estimate *M* can significantly contribute to these differences. For instance, Al-Beak et al. [81] estimated *M* using the equation proposed by Ursin [90], expressed as $M={\bar{W}}^{-1/3}$, where $\bar{W}$ represents the mean *W* of mature specimens in the sample. However, according to Pauly [91] and Höffle and Planque [92], this approach has long been abandoned and should be applied cautiously. Similarly, Villarta et al. [89] employed a semi-empirical, ratio-based method to estimate *M*, relying on the established relationship between *M* and growth parameter *K* [93,94].

The current *E* estimates derived from the mortality parameters obtained in this study were 0.63 for females and 0.69 for males, indicating a higher fishing impact on males. Both values exceeded the commonly accepted LRP of 0.5, clearly signaling overexploitation of the *P*. *semisulcatus* stock in the southeastern Red Sea. This finding aligns with all three previous investigations conducted in the Red Sea, which also reported *E* values surpassing the LRP threshold. Consistent patterns have been observed across much of the species’ distribution, with *E* estimates for other *P*. *semisulcatus* stocks ranging from 0.56 to 0.78 for females and 0.53 to 0.85 for males (Table 3). Particularly high *E* levels were documented by Maheswarudu et al. [86] in Palk Bay, India (0.78 for females and 0.85 for males), reflecting the intense fishing pressure in that region. In contrast, only a few studies have reported *E* values below or near the LRP of 0.5 (Table 3). Among the most recent of these are those by Bayhan [82] in the northeastern Mediterranean and Suman et al. [71] in Indonesian waters. Nevertheless, these few cases provide little evidence of a broader reduction in fishing pressure, and overall, the species appears to remain under high and unsustainable exploitation levels throughout its range.

According to the YpR estimates derived from the Thompson and Bell model for female *P*. *semisulcatus* (Figure 7), the current *F* of 3.75 year^−1^ substantially surpasses both *F*_0.1_ and *F_max_*, clearly indicating a situation of growth overfishing. This implies that female prawns are being harvested at a rate that hinders them from achieving their full growth potential, thereby reducing YpR and jeopardizing the long-term productivity of the stock. Additional evidence of growth overfishing, beyond the YpR analysis, is evident from the *CL* distribution of the sampled prawns. Almost 16% of the catch, a considerable portion, consisted of immature individuals (*CL* < *CL*_50_) during the study period (Figure 4). If this trend continues, further increases in fishing pressure are likely to lead to recruitment overfishing, which would compromise the reproductive capacity of the stock. The SPR analysis supports this concern, indicating that recruitment overfishing may already be occurring, as the current *F* has reduced the SSB to about 19% of its unfished level, well below both the TRP and LRP (Figure 7).

Two earlier studies, Sabry et al. [22] and Alsolami and Jastania [23], employed the length-based relative yield-per-recruit (Y′pR) analysis [51,95] to assess the stock status of *P*. *semisulcatus* along the southeastern Red Sea coast of Saudi Arabia. Since the input *F* array used in the traditional YpR analysis is replaced by an *E* array ranging from 0 to 1 in the Y′pR model, this approach yields two BRPs, *E*_0.1_ and *E_max_*, which correspond directly to the TRP of *F*_0.1_ and LRP of *F_max_* derived from the standard YpR model, respectively. According to Sabry et al. [22], the *E* estimated for females exceeded *E*_0.1_ and approached *E_max_*, whereas the value for males was approximately equal to *E*_0.1_. In contrast, Alsolami and Jastania [23] reported that the *E* estimated for both sexes combined surpassed *E_max_*, thereby supporting the present analysis and further demonstrating the severe overexploitation of *P*. *semisulcatus* in the region.

Several other studies employing Y′pR analysis have similarly reported *E* values exceeding the LRP *E_max_*, indicating overexploitation of *P*. *semisulcatus* stocks in various regions. These include the works of Yassien [7] and El-Ganainy and Yassien [72] in the Egyptian Mediterranean, Mehanna et al. [16] along the Omani coast of the Arabian Sea, Ragavan et al. [73] in Sri Lanka, and Rajkumar et al. [84] in Palk Bay, India. On the other hand, Abdul-Wahab [25] from the Yemeni Red Sea and Hassan et al. [17] from the northwestern Arabian Gulf concluded that the stocks they examined were not overexploited, as their estimated *E* values were below the TRP *E*_0.1_. However, it is noteworthy that the *E* estimates from both studies still exceeded the widely accepted LRP threshold of *E* = 0.5 (Table 3), suggesting that fishing pressure in these regions remains relatively high and potentially unsustainable, despite not surpassing *E*_0.1_.

To our knowledge, no previous study has applied an SPR-based stock assessment to *P*. *semisulcatus* in the Red Sea. Globally, SPR estimates for this species are also scarce. Employing the length-based spawning potential ratio (LBSPR) method developed by Hordyk et al. [94], Ragavan et al. [73] reported an SPR value of 0.10 for *P*. *semisulcatus* in Sri Lankan waters, indicating that SSB has been reduced to approximately 10% of its unfished level, providing clear evidence of severe overfishing. In Palk Bay, India, Rajkumar et al. [84] used both LBSPR and Thompson and Bell approaches to estimate SPR values of 0.31–0.32 for females and 0.21–0.24 for males. Based on these comparisons, the SPR estimated in the present study (0.19) places the *P*. *semisulcatus* stock of the southeastern Red Sea between the conditions reported for Sri Lanka and India, but noticeably closer to the severely depleted Sri Lankan stock. This suggests that the stock in the Red Sea is experiencing substantial recruitment overfishing and requires urgent management intervention.

While the YpR and SPR analyses in this study concentrated solely on females because their reproductive contribution, through egg production, plays a central role in determining overall stock productivity, it is also reasonable to extend this assessment to males. Male prawns experience the same fishing pressures as females, and their removal may indirectly impact reproductive performance through shifts in sex ratios and associated mating dynamics. A comprehensive assessment of the current levels of *E* and *F* relative to the chosen BRPs clearly shows that *P*. *semisulcatus* in the southeastern Red Sea is being harvested beyond sustainable limits. The prevailing fishing intensity has led to overexploitation, jeopardizing the stock’s long-term viability and emphasizing the urgent need for immediate management actions. It is strongly recommended to significantly reduce *F* to levels corresponding to either *F*_40%_ or *F*_0.1_, which requires lowering overall fishing effort by roughly half. This reduction may be accomplished through measures such as adjusting the number of permitted fishing days, limiting the active trawling fleet, or prolonging the current five-month shrimp-trawling closure in the region to enhance protection during the peak spawning and recruitment periods of *P*. *semisulcatus*.

Modifying gear selectivity could play a significant role in promoting fishery sustainability. Doll et al. [96] utilized Bayesian inference to demonstrate that when the length at first capture is significantly larger than the first maturity length, both SSB and yield can remain high, despite intense fishing activity. The commercial trawl gear used in the southeastern Red Sea shows a selectivity pattern that requires improvement, as almost 16% of the prawns sampled in this research were not mature (Figure 4). A recent study by Santucci et al. [32] in Jizan on selectivity of the trawl gear showed that immature *P*. *semisulcatus* were far more likely to escape from square-mesh codends than from the diamond-mesh versions used at present. Switching to square mesh codends would likely raise the current 14 mm *CL_c_*, which in turn would reduce the retention of small prawns that have not yet reached maturity and enhance the stock’s overall reproductive potential.

While the stock assessment results indicate that a reduction in fishing effort of at least 50% would theoretically be required to bring exploitation closer to sustainable levels (i.e., towards *F*_0.1_ or *F*_40%_), such an immediate decrease may not be feasible under current socioeconomic conditions. Accordingly, this value should be interpreted as a biological benchmark that reflects the magnitude of reduction needed to rebuild the stock, rather than as a prescriptive management target. In practice, progress toward sustainability can be achieved through incremental measures. A biologically grounded approach is to extend the current shrimp-trawl closure period (April–August) to include March, identified in this study as the onset of the major spawning peak. Additionally, introducing a one-month break in December, when a pronounced recruitment pulse was observed, would offer further protection to critical life stages. Extending the closure by these two months would shorten the effective trawl fishing season from seven to five months, resulting in an estimated 29% reduction in annual fishing effort (assuming effort is uniformly distributed across months). Under this scenario, *F* would be reduced from 3.75 year^−1^ to approximately 2.68 year^−1^, a value slightly lower than *F_max_* (2.72 year^−1^) derived from the Thompson and Bell analysis. Consequently, SPR would increase from 0.19 to roughly 0.25, and the SSB would rise to about one-quarter of the unfished level. Importantly, the YpR model indicates that such a reduction in effort would not diminish overall yield; instead, catch per unit effort is likely to increase, reducing fuel consumption and sea time while improving profitability. Once improvements in stock condition and fishery performance become evident, additional incremental reductions in fishing effort can be implemented to gradually move the stock towards more precautionary reference points (*F*_0.1_ or *F*_40%_) and support long-term rebuilding of the SSB.

## 5. Conclusions

This study presents an updated and detailed evaluation of the *P*. *semisulcatus* fishery in the southeastern Red Sea, integrating estimates of essential population parameters along with an appraisal of stock status. Within the study region, *P*. *semisulcatus* represented the predominant component of the shrimp catch, comprising more than 83% of the total samples. The findings from this initial assessment demonstrate that trawling pressure along the Saudi Arabian Red Sea coast currently exceeds biologically sustainable levels, clearly indicating that the stock is overexploited. Beyond the stock assessment metrics and BRPs indicating ongoing overfishing, the presence of a considerable proportion of immature prawns in the samples strongly suggests inadequate gear selectivity. Together, these results underscore the need for targeted management measures to curb fishing pressure and ensure the long-term sustainability of both the shrimp stocks and the fishery in the region. Considering its substantial economic importance, implementing effective and science-based management strategies is vital to safeguard the shrimp fishery’s future. Enhancing sustainability will require adopting square mesh codends with improved size selection properties that minimize the retention of immature prawns. Complementary measures may include input and output controls, such as limiting the number of fishing days, restricting the active trawling fleet, implementing catch limits, and prolonging the current five-month trawling closure to enhance conservation during key spawning and recruitment periods. Furthermore, maintaining robust and consistent data collection programs is essential for ongoing monitoring and adaptive management

Incorporating environmental variables into future modeling frameworks is crucial for achieving a more holistic comprehension of population dynamics. Considering the potential influence of climate change on marine resources, upcoming studies should integrate climate-driven environmental changes into management planning to promote the long-term viability of the *P*. *semisulcatus* fishery. This includes routine monitoring of sea surface temperature, salinity, dissolved oxygen, and habitat conditions, as well as tracking potential shifts in spawning seasonality, recruitment timing, and distribution patterns. Such information can support climate-responsive management measures, including adjusting seasonal closures, refining reference points, and developing adaptive harvest strategies. All recommended measures and monitoring activities should be integrated into a multi-year, ecosystem-based management framework created in partnership with all key stakeholders. This framework would provide a coordinated structure for applying management regulations, bolstering monitoring and data collection programs, and enabling adaptive decision-making through regular assessments of stock status and environmental conditions.

## Figures and Tables

**Table 1:** Summary statistics for carapace length (*CL*) and weight (*W*) of *P*. *semisulcatus* sampled from the southeastern Red Sea, reported for the full dataset and each sex separately. *n*, SD, and IQR refer to sample size, standard deviation, and interquartile range, respectively.

Sex	*n*	Carapace Length (mm)				Weight (g)			
		Range	Mean ± SD	Median	IQR	Range	Mean ± SD	Median	IQR
Female	41,657	2.03–56.14	30.54 ± 6.40	30.60	25.58–35.40	1.28–94.99	24.11 ± 12.34	22.64	13.88–32.56
Male	44,225	2.17–46.91	24.93 ± 3.27	25.00	22.40–27.28	1.45–62.64	13.91 ± 4.76	13.69	9.91–17.17
Total	85,909	1.29–56.14	27.65 ± 5.77	26.59	23.50–30.88	0.91–94.99	18.85 ± 10.56	15.95	11.32–23.18

**Table 3:** Summary of von Bertalanffy growth parameters (*CL*_∞_ and *K*), total mortality (*Z*), natural mortality (*M*), fishing mortality (*F*), and exploitation rates (*E*) of *P*. *semisulcatus* reported from different regions worldwide. F and M denote females and males, respectively. Values representing both sexes combined are placed between the F and M columns. An asterisk (*) indicates measurements taken as *TL* instead of *CL*.

Location	*CL*_∞_ (mm)		*K* (Year^−1^)		*M* (Year^−1^)		*F* (Year^−1^)		*Z* (Year^−1^)		*E*		References
	F	M	F	M	F	M	F	M	F	M	F	M	
Saudi Arabia (Red Sea)	60.2	48.1	1.03	1.02	2.21	2.27	3.75	4.99	5.96	7.26	0.63	0.69	Present study
	94.4		0.81		1.16		1.96		3.12		0.63		Alsolami and Jastania [23]
	58.5	44.9	0.8	0.7	1.65	1.59	2.29	2.01	3.94	3.60	0.58	0.55	Sabry et al. [22]
Saudi Arabia (Arabian Gulf)	57		1.9		2.39								Rabaoui et al. [18]
	62.0	51.5	1.10	1.77	1.47	2.12	1.90	2.53	3.37	4.65	0.56	0.54	Alrashada et al. [19]
Yemen (Red Sea)	58.8	44.6	1.4	1.2	2.27	2.19	3.37	4.36	5.63	6.55	0.60	0.67	Abdul-Wahab [25]
Yemen (Arabian Sea)	62	51	1.5	1.6	2.4	2.6	3.2	4.7	5.6	7.3	0.57	0.64	Abdul-Wahab [80]
Egypt (Red Sea)	268.4 *	224.2 *	1.56	1.77									Mehanna [74]
	104.3 *		1.84		3.64		5.28		8.64		0.61		El-Ganainy and Yassien [72]
Oman (Arabian Sea)	63.6	58.2	1.69	1.8	2.39	2.11	7.28	5.73	9.67	7.84	0.75	0.73	Mehanna et al. [16]
Kuwait (Arabian Gulf)	48.0	39.5	1.69	1.33	2.4		4.9		7.7		0.36		Siddeek [10]
	51.2	36.6	1.7	1.6	2.4	2.5	2.3	1.7	4.7	4.2	0.50	0.40	Mohammed et al. [12]
	51.3	36.6	1.94	1.64	2.20	2.52	4.57	5.66	6.77	8.18	0.68	0.69	Ye et al. [87]
Iraq (Arabian Gulf)	287		0.50		1.01		1.8		2.81		0.64		Hassan et al. [17]
Iran (Arabian Gulf)	50.4	38.0	2.2	1.6	2.41	2.11	5.8	4.3	8.2	6.4	0.70	0.67	Niamaimandi et al. [14]
	57.0	46.0	1.8	1.6	2.14	2.10	3.36	3.69	5.50	5.79	0.61	0.64	Alizadeh et al. [8]
	69.0	55.0	2.1	1.7	2.24	2.08	3.81	6.47	6.05	8.55	0.63	0.76	
Egypt (Mediterranean Sea)	221.5 *	197.2 *	1.0	1.1	1.79	1.96	4.46	5.43	6.25	7.39	0.71	0.73	Yassien [7]
	66.7	53.5	1.1	0.92	1.16	1.05	4.17	2.18	5.34	3.24	0.78	0.67	Mohamed and El-Aiatt [75]
	293.2 *	227.6 *	0.14	0.21	0.42	0.43	0.59	0.36	1.01	0.79	0.58	0.46	Al-Beak et al. [81]
Türkiye (Mediterranean Sea)	63	42	1.9	1.8	1.97	1.5	4.42	2.24	6.39	4.34	0.69	0.65	Manaşırlı et al. [77]
	262.5 *	199.5 *	0.8	1.3	1.02	2.12	0.57	2.22	1.59	4.34	0.36	0.51	Bayhan [82]
India (Arabian Sea)	248.8 *	218.4 *	1.5	1.55	2.3	2.38	5.15	5.57	8.45	8.91	0.61	0.63	CMFRI [83]
India (Bay of Bengal)	273 *	227 *	1.5	1.6	2.3	2.5	5.2	6.5	7.5	9	0.69	0.72	CMFRI [83]
	261 *	210 *	1.3	1.7									Rao et al. [88]
	279.1 *	231.4 *	1.16	1.34	1.78	2.06	6.36	11.87	8.14	13.93	0.78	0.85	Maheswarudu et al. [86]
	270.5 *	229.3 *	1.14	1.24	2.00	2.21	3.26	3.09	5.47	5.30	0.59	0.58	Rajkumar et al. [84]
Bangladesh (Bay of Bengal)	270 *	235 *	0.9	0.8	1.72	1.73	2.98	3.47	4.70	5.20	0.63	0.67	Mustafa et al. [78]
Sri Lanka (Bay of Bengal)	52.9		1.8		3.66		7.14		10.8		0.66		Ragavan et al. [73]
Indonesia	58.9		1.7		1.45		1.12		2.57		0.44		Suman et al. [71]
Philippines	271 *	263 *	1.6	0.7	3.65	1.70	2.00	1.91	5.65	3.61	0.35	0.53	Villarta et al. [89]

## Data Availability

The data presented in this study are available upon request from the corresponding author (the data are not publicly available due to privacy restrictions set by the funding institution).

## Supplementary Materials

The following supporting information can be downloaded at: https://www.mdpi.com/article/10.3390/biology15010008/s1, Figure S1: Measurement of carapace length from the posterior margin of the orbit to the posterior end of the mid-dorsal line of the carapace using a Vernier caliper; Table S1: Description of gonadal maturity stages (I–IV) in female *Penaeus semisulcatus*, including stage classification, category, and corresponding ovarian characteristics; Figure S2: Ovarian maturation stages in *Penaeus semisulcatus*: (a) Stage I—Immature or undeveloped; (b) Stage II—Developing; (c,d) Stage III—Mature; (e,f) Stage IV—Spent; Figure S3: Monthly variations in the percentage sex ratio of *P*. *semisulcatus* in the southeastern Red Sea; Figure S4: (a) Monthly carapace length frequency distributions of male *P*. *semisulcatus*, arranged in 2 mm size classes; (b) the same distributions smoothed and restructured using a 5-class moving average, shown together with the seasonally oscillating von Bertalanffy growth curves (dashed maroon lines). Both visualizations were produced with the TropFishR package; Figure S5: (a) Monthly carapace length frequency distributions of female *P*. *semisulcatus*, arranged in 2 mm size classes; (b) the same distributions smoothed and restructured using a 5-class moving average, shown together with the seasonally oscillating von Bertalanffy growth curves (dashed maroon lines). Both visualizations were produced with the TropFishR; Figure S6: Linearized length-converted catch curve plots used to estimate the annual total mortality rate (*Z*) for *P*. *semisulcatus*, based on simple linear regression: (a) males and (b) females. CI indicates the confidence interval associated with *Z*. The gray shading around regression lines illustrates the corresponding 95% confidence bands.
