# Supplementary material for: Linking Biological Parameters to Fishery Management: Stock Assessment of Green Tiger Prawn, Penaeus semisulcatus De Haan, 1844 Along the Red Sea Coast of Saudi Arabia
Source: Biology (Basel). 2025 Dec 19;15(1):8. doi: 10.3390/biology15010008 (PMC12784927; doi:10.3390/biology15010008)
Supplement: Supplementary file 1 [file biology-15-00008-s001.zip › biology-3996910-supplementary.pdf]

biology

Supplementary materials

MDPI

Article

Linking Biological Parameters to Fishery Management: Stock Assessment of Green Tiger Prawn, Penaeus semisulcatus De Haan, 1844 Along the Red Sea Coast of Saudi Arabia

Eyüp Mümtaz Tıraşın [1,2], Sheeja Gireesh [1], Sirajudheen Thayyil Kadengal [1], Ronald Grech Santucci [1], Zahra Okba [1,*], Santhosh Kumar Charles [1], Goutham Bharathi Muthu Palani [1], Adel M. S. Adam [1], and Mark Dimech [1]

1 KAUST Beacon Development Department, National Transformation Institute, King Abdullah University of Science and Technology, Innovation Cluster, 4700, 23955-6900, Thuwal, Saudi Arabia; mumtaz.tirasin@deu.edu.tr (EMT); sheeja.gireesh@kaust.edu.sa (S.G.); sirajudheen.kadengal@kaust.edu.sa (S.T.K.); ronald.grechsantucci@kaust.edu.sa (R.G.S.); santhosh.charles@kaust.edu.sa (S.K.C.); goutham.muthupalani@kaust.edu.sa (G.B.M.P.); adel.adam@kaust.edu.sa (A.M.S.A.); mark.dimech@kaust.edu.sa (M.D.)
2 Institute of Marine Sciences and Technology, Dokuz Eylül University, İnciraltı 35340, İzmir, Türkiye
* Correspondence: zahra.okba@kaust.edu.sa

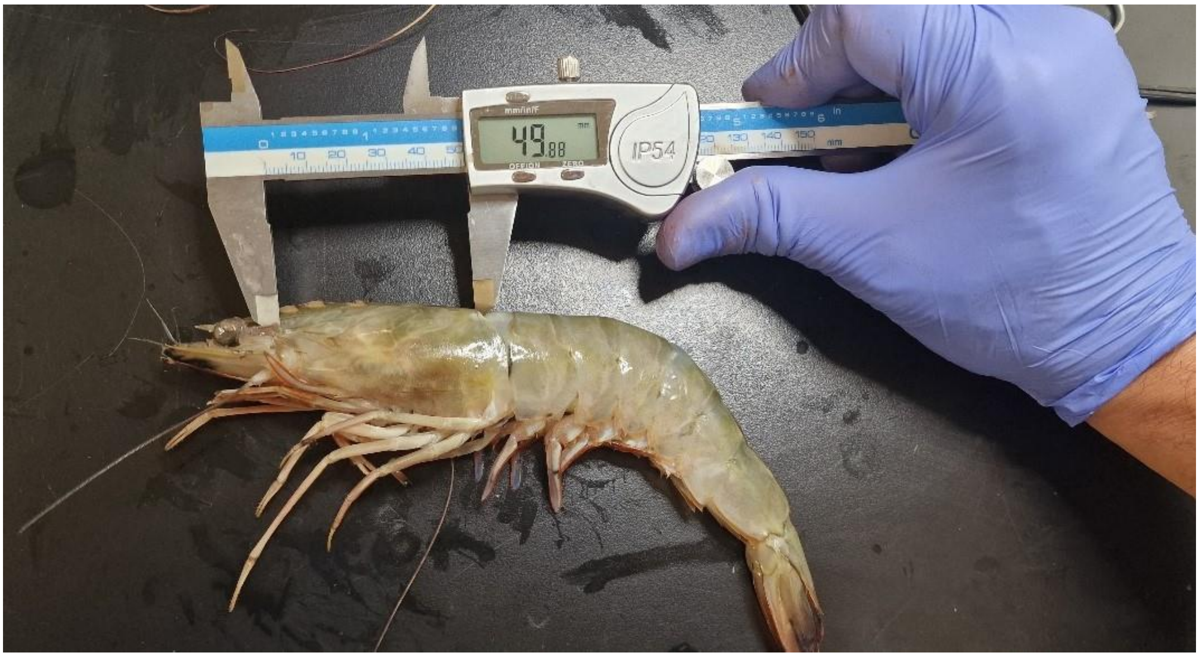

Figure S1. Measurement of carapace length from the posterior margin of the orbit to the posterior end of the mid-dorsal line of the carapace using a Vernier caliper.

Table S1. Description of gonadal maturity stages (I–IV) in female *Penaeus semisulcatus*, including stage classification, category, and corresponding ovarian characteristics.

Maturity Stages	Category	Ovarian characteristics
Stage I	Immature/ undeveloped	Ovaries are thin, translucent, and underdeveloped, with poorly defined anterior and lateral lobes, not visible through the exoskeleton
Stage II	Developing	Ovaries appear light yellow-green and are larger in size, and in more advanced cases, within this stage, they occupy most of the dorsal portion, light green in color, and become visible through the dorsal exoskeleton
Stage III	Mature	Ovaries are fully developed, occupying the entire dorsal region, are dark green in color, and are visible through the exoskeleton
Stage IV	Spent	Ovaries resemble those in Stage II in color but are more enlarged, flaccid in texture, and reduced in turgidity, either slightly visible or no longer visible through the exoskeleton

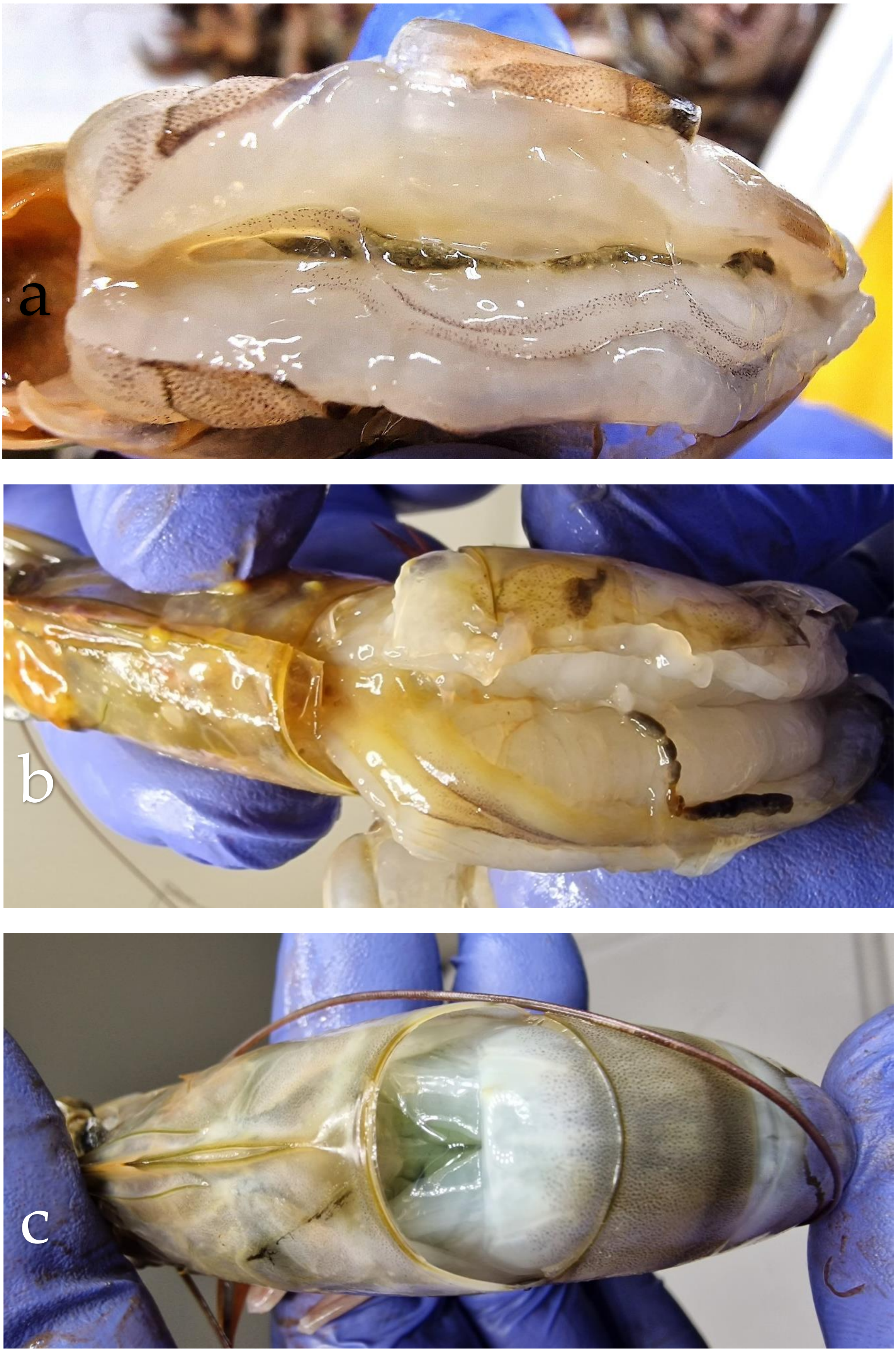
a
b
c

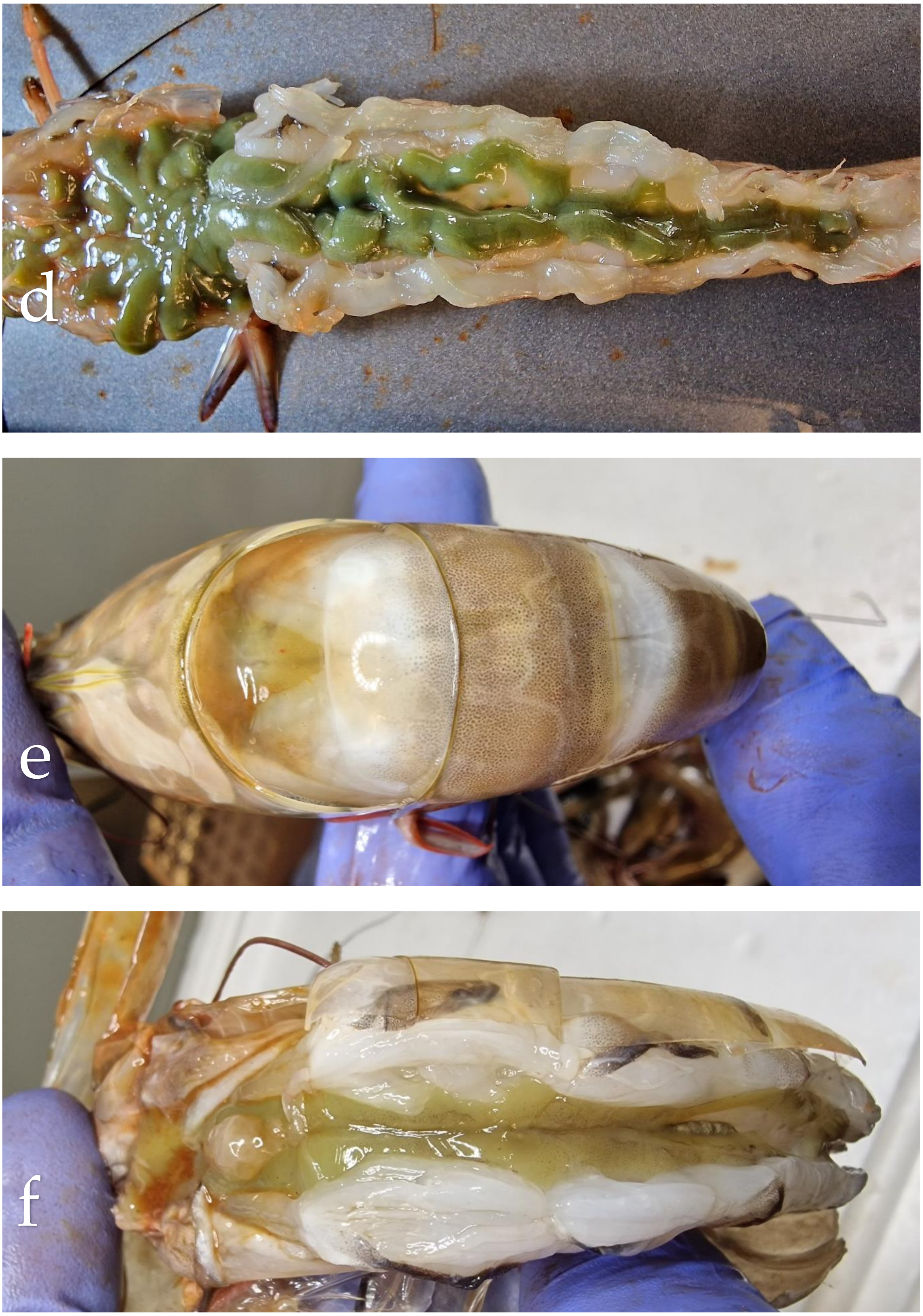

Figure S2. Ovarian maturation stages in *Penaeus semisulcatus*: (a) Stage I—Immature or undeveloped; (b) Stage II—Developing; (c, d) Stage III—Mature; (e, f) Stage IV—Spent.

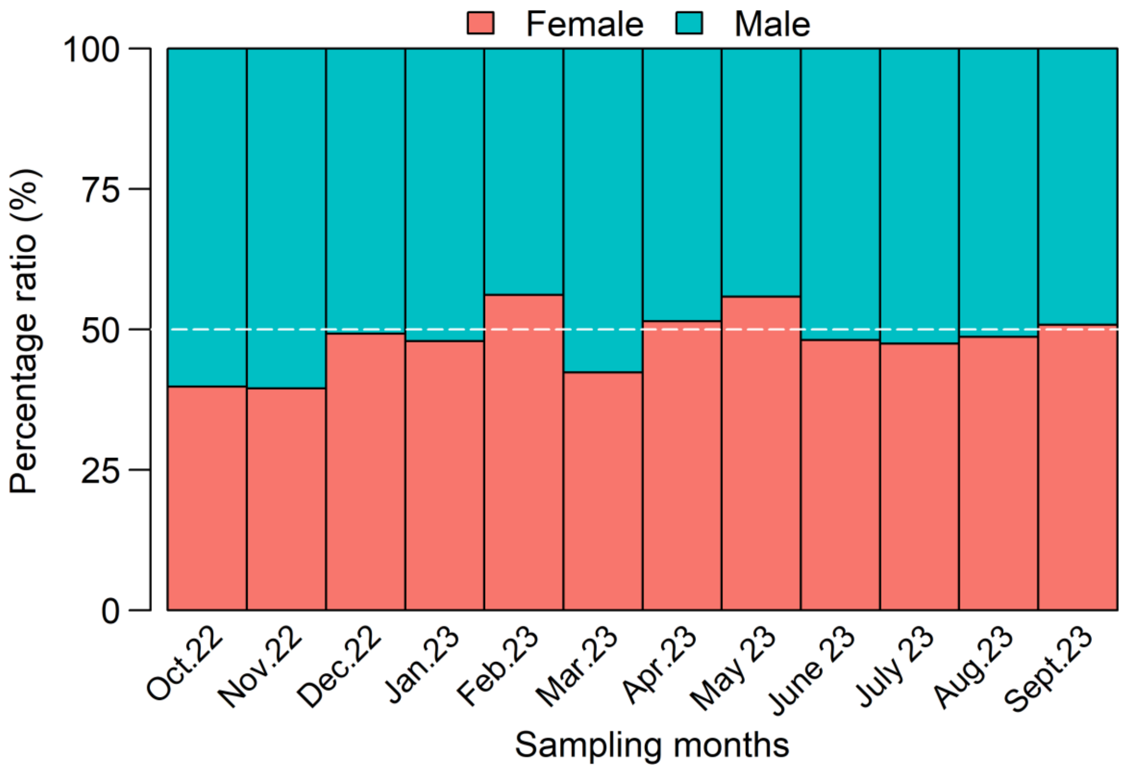

Figure S3. Monthly variations in the percentage sex ratio of *P. semisulcatus* in the southeastern Red Sea.

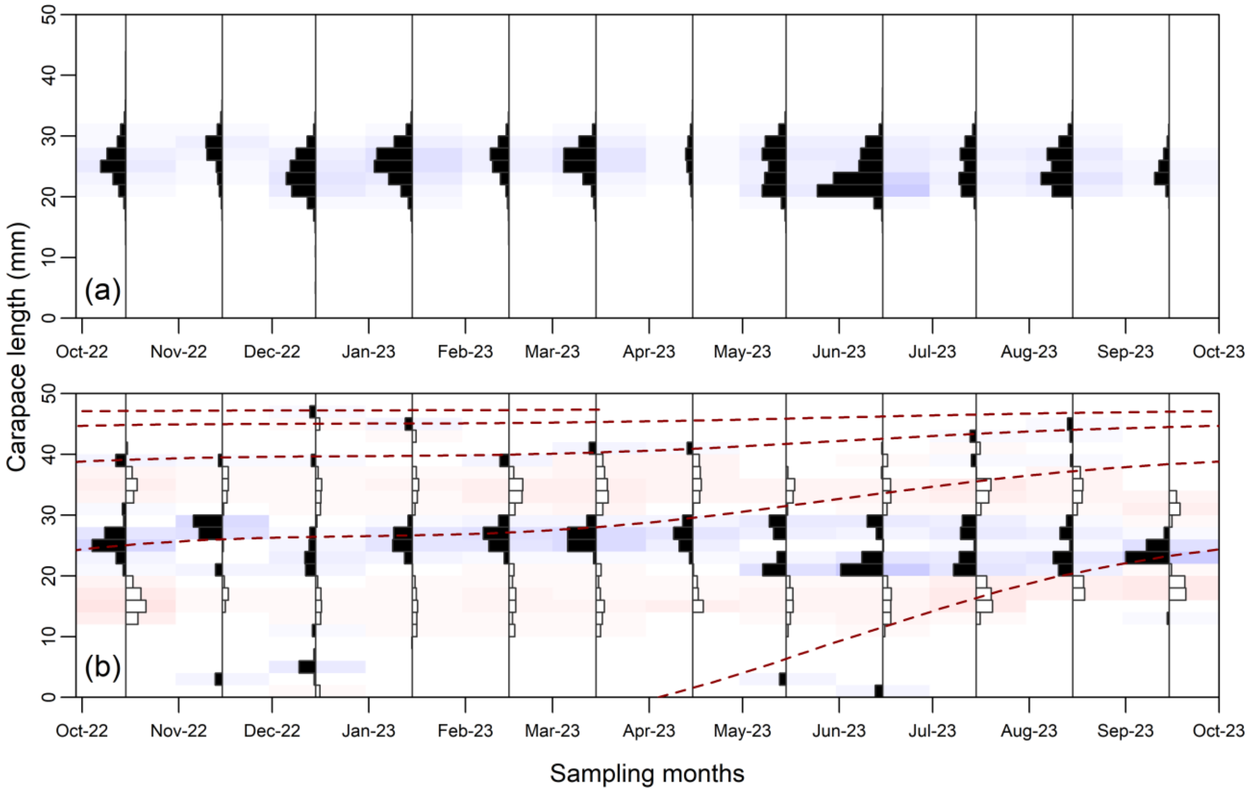

Figure S4. (a) Monthly carapace length frequency distributions of male *P. semisulcatus*, arranged in 2-mm size classes; (b) the same distributions smoothed and restructured using a 5-class moving average, shown together with the seasonally oscillating von Bertalanffy growth curves (dashed maroon lines). Both visualizations were produced with the TropFishR package.

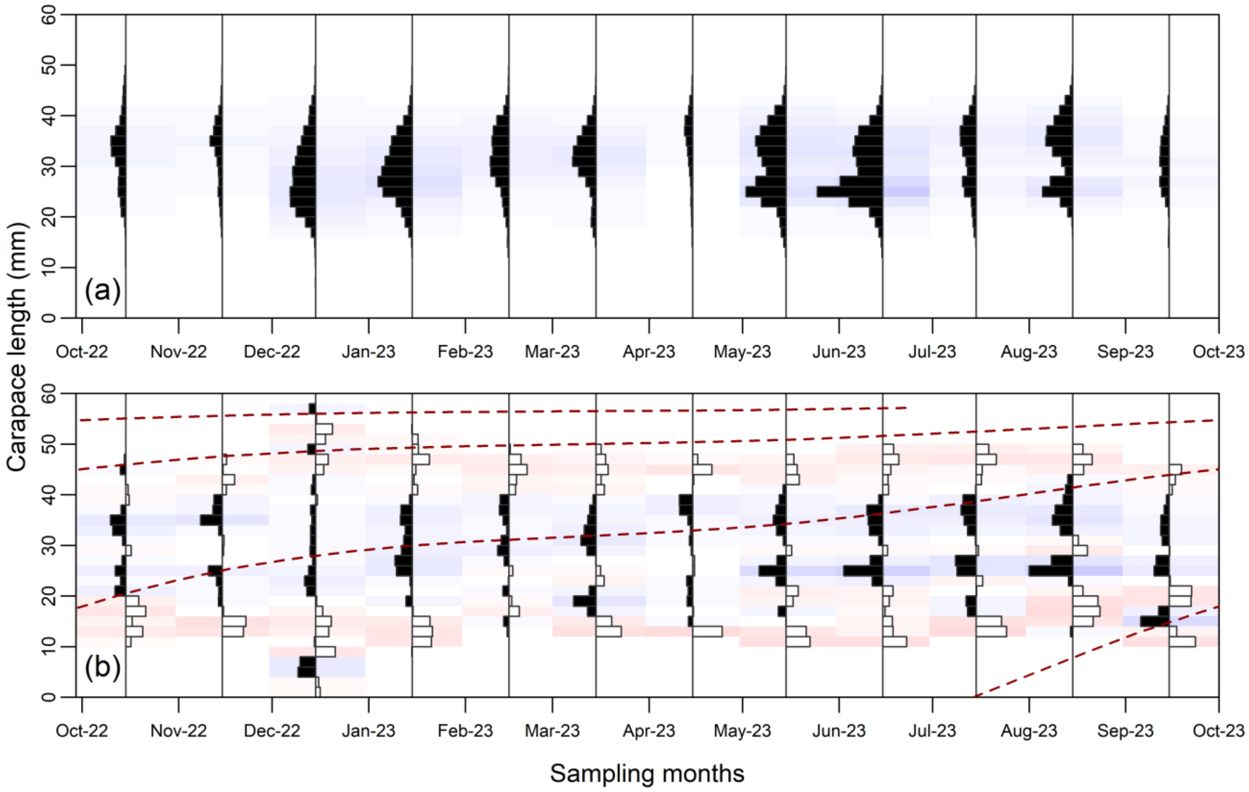

Figure S5. (a) Monthly carapace length frequency distributions of female *P. semisulcatus*, arranged in 2-mm size classes; (b) the same distributions smoothed and restructured using a 5-class moving average, shown together with the seasonally oscillating von Bertalanffy growth curves (dashed maroon lines). Both visualizations were produced with the TropFishR package.

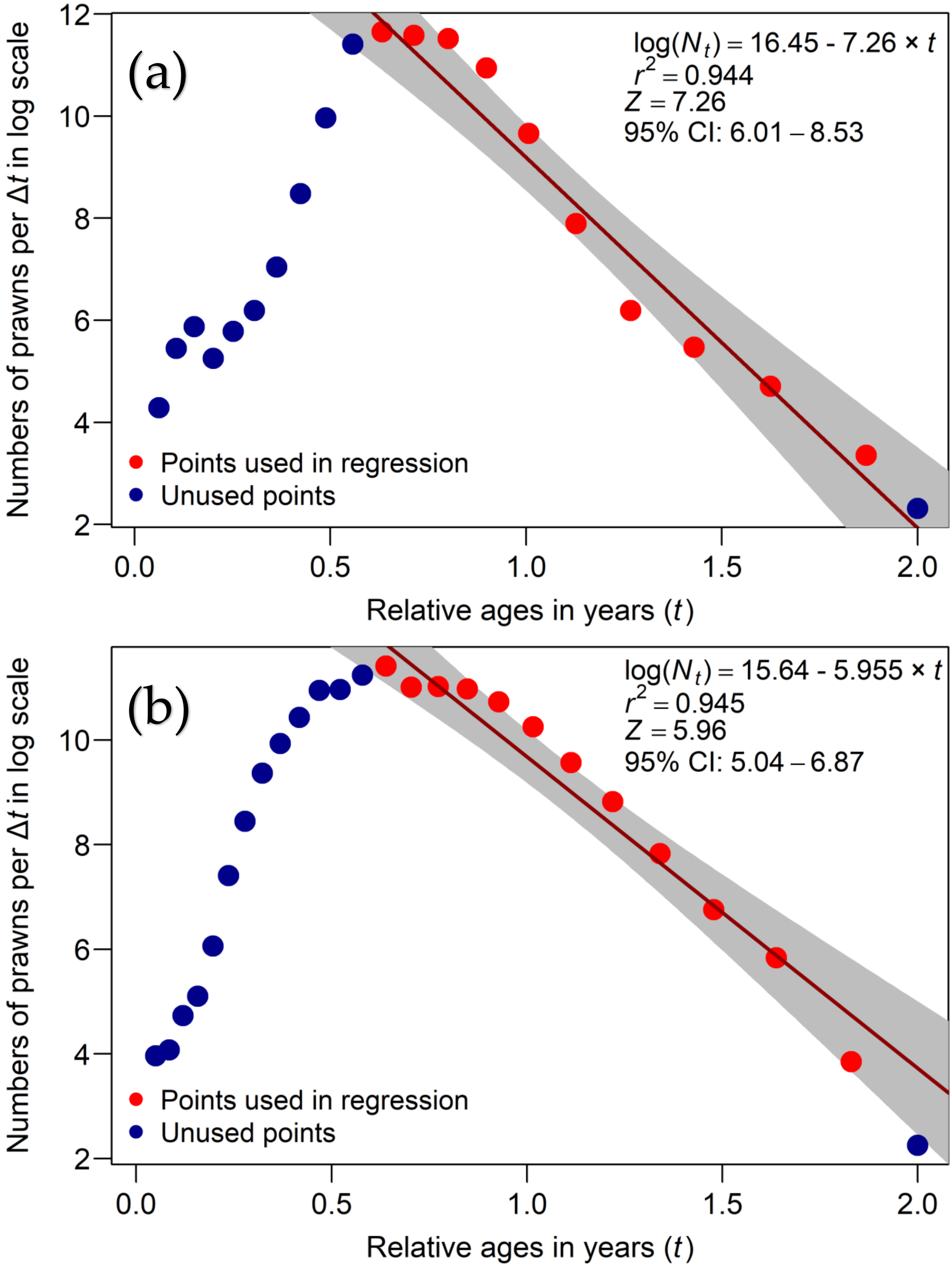

Figure S6. Linearized length-converted catch curve plots used to estimate the annual total mortality rate (Z) for *P. semisulcatus*, based on simple linear regression: (a) males and (b) females. CI indicates the confidence interval associated with Z. The grey shading around regression lines illustrates the corresponding 95% confidence bands.
